# Real-Time Spiral CMR Is Superior to Conventional Segmented Cine-Imaging for Left-Ventricular Functional Assessment in Patients with Arrhythmia

**DOI:** 10.3390/jcm11082088

**Published:** 2022-04-08

**Authors:** Nicoleta Nita, Johannes Kersten, Alexander Pott, Fabian Weber, Temsgen Tesfay, Marius-Tudor Benea, Patrick Metze, Hao Li, Wolfgang Rottbauer, Volker Rasche, Dominik Buckert

**Affiliations:** 1 Department of Internal Medicine II, University Medical Center, 89081 Ulm, Germany; johannes.kersten@uniklinik-ulm.de (J.K.); alexander.pott@uniklinik-ulm.de (A.P.); fabian.weber@uni-ulm.de (F.W.); temsgen.tesfay@uniklinik-ulm.de (T.T.); patrick.metze@uni-ulm.de (P.M.); hao.li@uni-ulm.de (H.L.); wolfgang.rottbauer@uniklinik-ulm.de (W.R.); volker.rasche@uni-ulm.de (V.R.); dominik.buckert@uniklinik-ulm.de (D.B.); 2 Faculty of Medicine, Lucian Blaga University, 550169 Sibiu, Romania; tudor.benea@ulbsibiu.ro

**Keywords:** real-time CMR, spiral real-time CMR, CMR volumetry in arrhythmia, CMR in atrial fibrillation

## Abstract

(1) Background: Segmented Cartesian Cardiovascular magnetic resonance (CMR) often fails to deliver robust assessment of cardiac function in patients with arrhythmia. We aimed to assess the performance of a tiny golden-angle spiral real-time CMR sequence at 1.5 T for left-ventricular (LV) volumetry in patients with irregular heart rhythm; (2) Methods: We validated the real-time sequence against the standard breath-hold segmented Cartesian sequence in 32 patients, of whom 11 presented with arrhythmia. End-diastolic volume (EDV), end-systolic volume (ESV), stroke volume (SV), and ejection fraction (EF) were assessed. In arrhythmic patients, real-time and standard Cartesian acquisitions were compared against a reference echocardiographic modality; (3) Results: In patients with sinus rhythm, good agreements and correlations were found between the segmented and real-time methods, with only minor, non-significant underestimation of EDV for the real-time sequence (135.95 ± 30 mL vs. 137.15 ± 31, *p* = 0.164). In patients with arrhythmia, spiral real-time CMR yielded superior image quality to the conventional segmented imaging, allowing for excellent agreement with the reference echocardiographic volumetry. In contrast, in this cohort, standard Cartesian CMR showed significant underestimation of LV-ESV (106.72 ± 63.51 mL vs. 125.47 ± 72.41 mL, *p* = 0.026) and overestimation of LVEF (42.96 ± 10.81% vs. 39.02 ± 11.72%, *p* = 0.039); (4) Conclusions: Real-time spiral CMR improves image quality in arrhythmic patients, allowing reliable assessment of LV volumetry.

## 1. Introduction

Cardiovascular magnetic resonance (CMR) is a well-established method for functional cardiac assessment and plays a pivotal role in modern diagnosis and clinical management of cardiovascular diseases [1,2]. However, volumetry analysis in patients with arrhythmias remains challenging using standard segmented cine acquisitions with balanced steady state free precession (b-SSFP), considerably limiting diagnostic accuracy in this increasing population. Real-time CMR techniques attempt to overcome these limitations; to date, various approaches using combinations of sampling and reconstruction techniques [3,4,5,6,7,8] as well as parallel imaging [9,10] have been implemented, albeit with inferior diagnostic performance due to reduced temporal and/or spatial resolution. Volumetric assessment has been validated in patients with sinus rhythm for different real-time cine sequences at 1.5 [5,11,12] and 3 tesla (T) [8,13], yet arrhythmic patients have been poorly represented. Moreover, many studies reported systematic underestimation of end-diastolic volume (EDV) due to insufficient coverage of cardiac cycle in prospective ECG triggering real-time CMR for data acquisition [8,11,14].

In this study, we investigated the application of a tiny golden-angle b-SSFP spiral sequence for real-time cardiac imaging with the objective to validate its performance in direct comparison with the breath-hold segmented standard sequence for left-ventricular (LV) volumetry in patients with arrhythmias.

## 2. Materials and Methods

### 2.1. Study Population

This prospective study enrolled ambulant or hospitalized patients referred to CMR examination in our institution for various clinical indications from March 2019 to September 2021 and who consented for additional research scans. The study included 32 consecutive patients, of whom 21 had sinus rhythm. Eleven patients had arrhythmia in form of atrial fibrillation (nine patients) and ventricular extrasystoles (two patients) confirmed by electrocardiogram (ECG). Patients who had cardiac pacemakers, defibrillators, non-MRI compatible implants, or claustrophobia at the time of scanning were excluded.

The study protocol was approved by the ethics committee of the University of Ulm and all participants provided informed consent in accordance with Declaration of Helsinki.

### 2.2. CMR Protocol

All CMR scans were performed on a clinical 1.5 T magnetic resonance scanner (Achieva, Philips Healthcare, Best, The Netherlands). Acquisitions were performed in supine position using a 32-channel phased-array cardiac coil (Philips Healthcare), and a vector electrocardiographic system was used for cardiac gating. The investigation was part of the conventional CMR protocol, and the real-time sequences were additionally performed at the end of the standard acquisitions.

A set of 2D b-SSFP cine acquisitions was performed in two, three-, and four-chamber orientations followed by a stack of short-axis 2D b-SSFP cine slices spanning the entire left ventricle from the base to the apex. These scans were obtained with two sequences in each patient: segmented Cartesian acquisition and real-time spiral acquisition. Slice orientation, thickness, and interslice were the same for each sequence.

Segmented Cartesian acquisition: Standard cine CMR was performed using a multi-slice b-SSFP Cartesian sequence. Retrospective ECG-gating was used to cover the entire cardiac cycle with 32 phases, leading to a temporal resolution of ~30 ms, depending on the heart rate. Contiguous slices were acquired in the short axis to allow full coverage of the ventricle (~11 ± 2 slices, range: 11 to 17). Each slice was acquired in ~4 ± 0.69 s (3 slices for 1 breath hold). CMR images were acquired during breath-holding in end-expiration. Detailed sequence parameters are presented in Table 1.

Real-time spiral CMR acquisition and reconstruction: Real-time b-SSFP imaging was performed using a modified uniform density Archimedean spiral sequence, as described by Wundrak et al. [15], with a tiny golden-angle angular increment of $\psi_{7}=23.62814{}^{\circ}$, a fixed readout length of 3 ms, and a compromised spatial resolution of 2 × 2 × 8 mm^3^. A rewinder gradient was applied at the end of the spiral readout to maintain b-SSFP coherence. For each slice, data were acquired continuously for 3 s during free breathing over at least two R–R intervals, with the first R–R interval used to reach steady-state condition and the second for the actual image data acquisition. Data were reconstructed from seven consecutively acquired spiral readouts, yielding a real temporal resolution of 39.5 ms. Depending on the required number of slices for full LV coverage, the real-time sequences required an additional scan time of 44 s (11 slices)–68 s (17 slices). To address aliasing artifacts introduced by the 5-fold undersampling, all data were reconstructed offline by a k–t SPARSE–SENSE framework with a Total Variation (TV) sparsity operator, according to Wundrak et al. [16], and implemented in MATLAB (MathWorks, Natick, MA, USA).

### 2.3. Image Quality Assessment and CMR Data Analysis

Two experienced cardiologists with board certification in cardiovascular MRI performed initial qualitative assessment of cine images before further quantitative assessment. A standardized assessment of image quality was performed according to the European CMR registry criteria [17]. Higher scores are related to worse image quality due to increased artifact occurrence. Interobserver variability was measured for the qualitative evaluation of the cine data sets.

The quantitative assessment of left-ventricular ejection fraction (LVEF), end-systolic volume (ESV), end-diastolic volume (EDV), and stroke volume (SV) was performed for each patient by one certified observer using the Segment software (Medviso, v3.0 R7640, Lund, Sweden). For consistency and in accordance with recommendations of the Society for Cardiovascular Magnetic Resonance, the basal ventricular slice was defined as the plane in which 50% of the blood pool was surrounded by myocardium, and the most apical slice was determined as the last plane having blood pool at the end of diastole. The papillary muscles were not included in the calculation of LV mass quantification and were allocated to the ventricular cavity.

### 2.4. Echocardiographic Assessment

In patients with arrhythmia, both segmented and real-time-based CMR volumetry were additionally compared to echocardiography as a reference gold-standard volumetry method. In these 11 arrhythmic patients, quantitative assessment of ESV, EDV with derived LVEF, and SV was performed according to well-defined standards by a cardiologist certified in transthoracic echocardiography by the European Association of Cardiovascular Imaging. Volumetry measurements followed the biplane method of discs (modified Simpson’s rule), using apical four-chamber and two-chamber views. As for CMR and according to the recommendations of the European Society of Echocardiography, the papillary muscles and trabeculations were assigned to the LV cavity. Measurements were performed using TomTec 4DLV analysis software 2.7 (Image-Arena version 4.1; TomTec, Unterschleißheim, Germany).

### 2.5. Statistical Analysis

Statistical analyses were performed using IBM SPSS software (Version 25.0. IBM Corp.: Armonk, NY, USA), STATGRAPHICS Centurion (Version 19.2.02, STATGRAPHICS Technologies, The Plains, VA, USA), and G*Power (Version 3.1.9.6, Heinrich Heine University Düsseldorf, Düsseldorf, Germany).

Normality of the data was tested using the Shapiro–Wilk test. LV functional parameters derived from segmented Cartesian CMR, real-time CMR, and echocardiography were compared using paired-samples t-test or non-parametric Wilcoxon matched-pairs test. Bland–Altman analysis was performed among patients with sinus rhythm to assess agreement of the quantitative functional parameters measured by standard segmented method and spiral real-time method. The segmented data were used as the reference standard for Bland–Altman analysis. Proportional bias of volumetric variables was further evaluated using linear regression analysis. Inter-observer variability for the qualitative image scoring was assessed using a two-way intraclass correlation coefficient (ICC).

Within the arrhythmic subgroup, additional analysis was performed to assess correlation, agreement, and equivalence between either the spiral or segmented method and the echocardiographic method defined as the reference standard for patients with arrhythmia. Correlation coefficients calculated using Pearson and Spearman methods were further supported by curve-estimation regression models. Additional two-sample equivalence tests were performed among arrhythmic patients to determine whether the means of either the segmented method or spiral method were equivalent with the means assessed by the reference echocardiographic method. Equivalence was demonstrated by showing that the 95% confidence interval for the difference between the means is entirely within the chosen margins of equivalence. A post hoc power analysis was also performed using G*Power, in order to assess the achieved power, for those two-tailed tests which rejected the null hypothesis. The analysis was performed based on the significance level of 0.05, the sample size considered for those cases, the correlation between the groups, and based on the effect size, determined from the difference between the dependent means of the two groups and their standard deviation, for the considered matched pairs.

All results are expressed as the mean ± standard deviation. A *p*-value of 0.05 or less was considered statistically significant.

## 3. Results

### 3.1. Patients’ Characteristics

A total of 32 patients were consecutively included in the study. Eleven patients had arrhythmia. The mean scan time of segmented Cartesian CMR was significantly longer than that of real-time spiral CMR (4 ± 0.69 s vs. 3 ± 0.27 s, *p*-value < 0.001). Real-time and standard segmented data sets were successfully acquired in all patients.

Patients’ demographic and clinical characteristics are summarized in Table 2. The mean age of the participants was 57 years and coronary artery disease was the main indication for performing the investigation. The mean heart rate during examination was 70 ± 11 beats/min for patients with sinus rhythm and 77 ± 8 beats/min for the arrhythmic participants.

### 3.2. Volumetry Assessment in Patients with Sinus Rhythms

**Figure 1:**
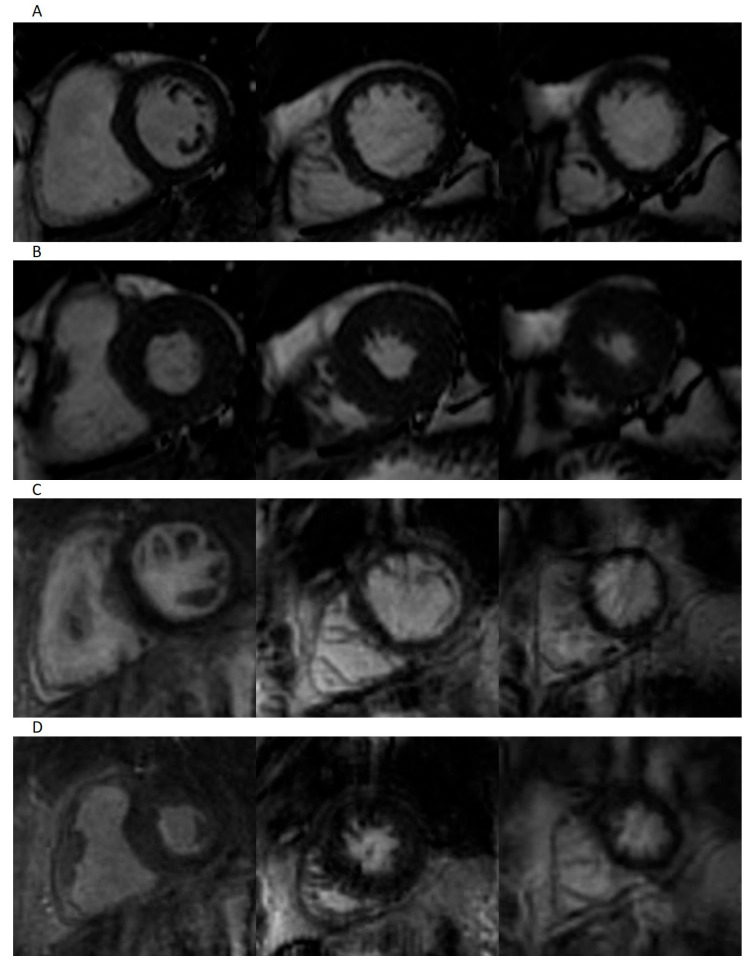
Representative images of patient in sinus rhythm. Short-axis-view images for segmented imaging in end-diastole (**A**) and end-systole (**B**), and spiral real-time imaging in end-diastole (**C**) and end-systole (**D**).

Both real-time and standard segmented acquisitions yielded high-quality images in patients with sinus rhythm, allowing accurate assessment of ventricular volumes. Real-time and segmented cine techniques performed comparably for image quality and noise, although basal slices obtained with real-time CMR showed a slightly inferior artifact performance compared with Cartesian CMR. Representative images are depicted in Figure 1, showing that spiral real-time acquisitions are of good quality. No significant differences between the Euro CMR scores for the two sequences could be noted for patients in sinus rhythm, as shown in Table 3. There was a high interrater reliability for the Euro CMR cumulative scoring between the two observers for both spiral real-time sequences (ICC 0.878, 95% CI 0.704 to 0.950) and segmented Cartesian sequences (ICC 0.911, 95% CI 0.783 to 0.964).

In this cohort, volumetry obtained with real-time spiral CMR was validated against the standard segmented Cartesian CMR defined as the reference technique for patients in sinus rhythm.

**Figure 2:**
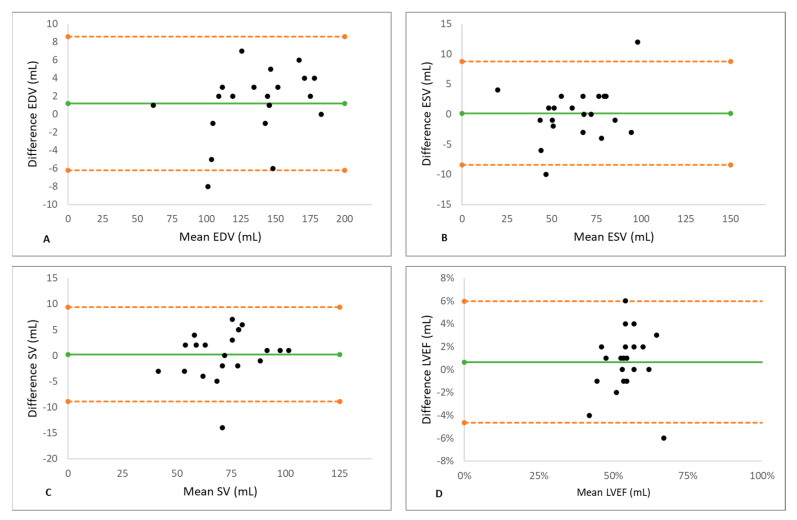
The Results of the Bland–Altman Analysis for the left ventricle. Good agreements for measurements of EDV (**A**), ESV (**B**), SV (**C**), and EF (**D**) by real-time CMR and segmented CMR were observed. Green line indicates mean value and orange dashed lines indicate the limits of agreement. No significant degree of directional measurement bias was observed for any parameter between spiral real-time CMR and segmented CMR. EDV = end-diastolic volume, EF = ejection fraction, ESV = end-systolic volume, SV = stroke volume, LV = left ventricle.

**Figure 3:**
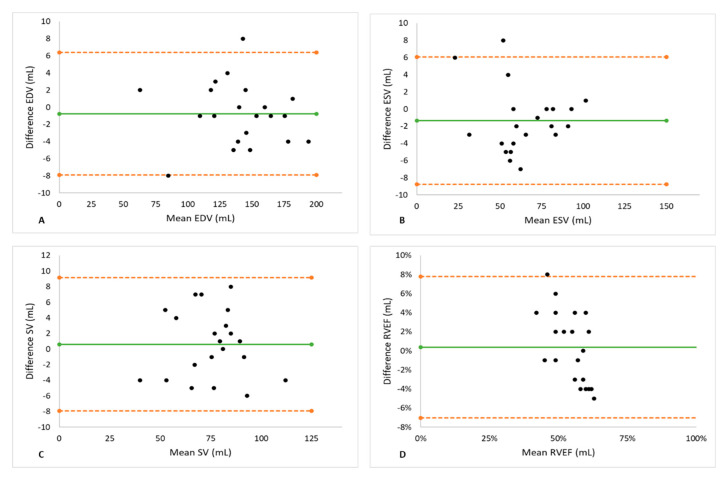
The Results of the Bland–Altman Analysis for the right ventricle. Good agreements for measurements of EDV (**A**), ESV (**B**), SV (**C**), and EF (**D**) by real-time CMR and segmented CMR were observed. Green line indicates mean value and orange dashed lines indicate the limits of agreement. No significant degree of directional measurement bias was observed for any parameter between spiral real-time CMR and segmented CMR. EDV = end-diastolic volume, EF = ejection fraction, ESV = end-systolic volume, RV = right ventricle, SV = stroke volume.

Biventricular metrics measured using spiral real-time and segmented Cartesian imaging are shown in Table 4. The two methods yielded similar results of EDV, ESV, SV, and EF for both ventricles. There was a small (1.2%), statistically non-significant underestimation of LV EDV (*p* = 0.164) using spiral acquisition. The Bland–Altman analysis for the LV showed good agreements between spiral real-time CMR and segmented CMR with no significant degree of directional measurement bias, as shown in Table 5 and Figure 2. Similar to the left ventricle, the Bland–Altman plots for the right ventricle (Figure 3) show narrow limits of agreement between the segmented and real-time acquisitions for all parameters. No proportional bias was indicated by the linear regression, with no mean coefficient (i.e., slope) significantly different from 0, according to Table 5.

### 3.3. Volumetry Assessment in Patients with Arrhythmia

**Figure 4:**
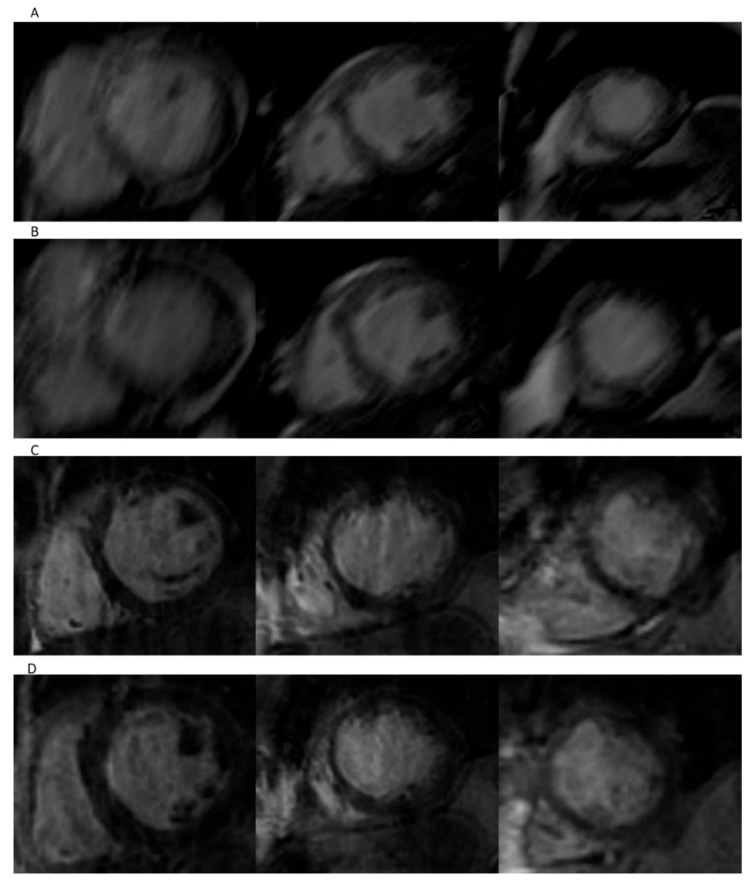
Representative images of patient in atrial fibrillation. Short-axis-view images for segmented imaging in end-diastole (**A**) and end-systole (**B**), and spiral real-time imaging in end-diastole (**C**) and end-systole (**D**).

Preliminary qualitative assessment showed more artifact burden in standard Cartesian cine imaging compared to spiral real-time cine method in the arrhythmic population. Representative images are depicted in Figure 4. The Euro CMR score showed a significantly higher artifact burden for the segmented sequence compared to the spiral real-time sequence, especially due to blurring and mis-triggering artifacts, as shown in Table 6. A high interrater reliability was calculated for the Euro CMR cumulative scoring of the two observers for both spiral real-time sequences (ICC 0.911, 95% CI 0.681 to 0.976) and segmented Cartesian sequences (ICC 0.986, 95% CI 0.894 to 0.997).

In arrhythmic patients, volumetry obtained with both segmented standard CMR and spiral real-time method were separately compared with echocardiographic volumetry defined as the reference standard.

Volumetry obtained using segmented Cartesian CMR showed significant underestimation of LV-ESV (*p*-value = 0.026) and significant overestimation of LVEF (*p*-value = 0.039). The post hoc power analysis performed for the cases in which the null hypothesis was rejected showed a statistical power higher than 98% (i.e., a probability of 0.989 for the first test and, respectively, of 0.999 for the second test), for the considered sample size of 11. The computed statistical power shows, therefore, a very high probability for the correct rejection of the null hypothesis, in both cases. A summary of the data obtained with segmented Cartesian CMR protocol and real-time spiral technique compared to the echocardiographic method is presented in Table 7. More importantly, spiral real-time acquisitions yielded similar results to the reference echocardiographic acquisitions, as only minor, statistically non-significant overestimation of EDV could be observed.

**Figure 5:**
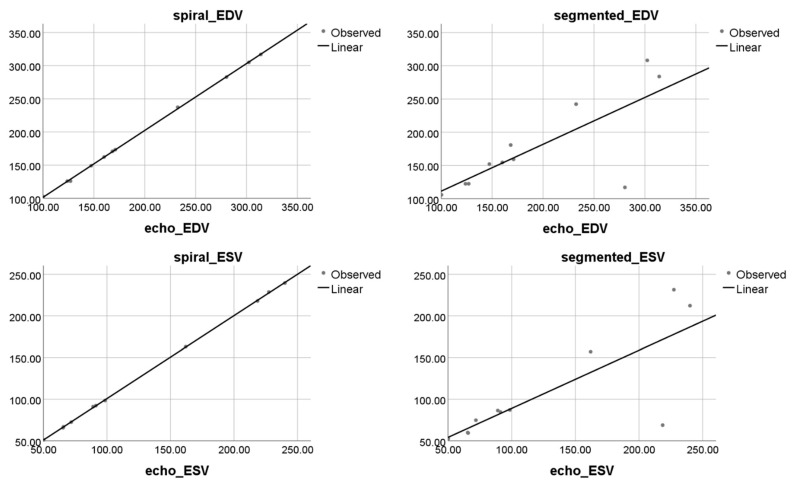
Results of linear regression analysis. Scatter plots on left side for LV volumetric measurements in spiral real-time CMR show strong agreement in EDV and ESV with measurements obtained using the reference echocardiographic method. In comparison, on the right side, segmented Cartesian CMR-based measurements for EDV and ESV show weaker agreement with the reference echocardiographic method.

Moreover, linear regression showed very strong correlation and agreement between real-time spiral CMR and echocardiographic acquisitions in EDV (y = 1.008x + 0.775, R^2^ = 1), ESV (y = 0.996x + 1.114, R^2^ = 1), EF (y = 1.002x + 0.341, R^2^ = 0.995), and SV (y = 1.005x − 0.381, R^2^ = 0.997), as shown in Figure 5. By comparison, linear regression showed weaker correlation and agreement between segmented CMR and echocardiographic acquisition EDV (y = 0.70x + 40.7, R^2^ = 0.58), ESV (y = 0.697x + 19.241, R^2^ = 0.632), EF (y = 0.816x + 11.11, R^2^ = 0.783), and SV (y = 1.091x − 5.934, R^2^ = 0.771) as shown in Figure 5. The two outliers depicted by the scatter plots of segmented Cartesian CMR confronted to echocardiographic data corresponded to a patient with dilative cardiomyopathy and a patient with ischemic cardiomyopathy. Both patients presented with atrial fibrillation, and the heart rate during the investigation was 77 beats/min and 83 beats/min, respectively. In both cases, the image quality of segmented CMR was poor and resulted in a significant underestimation of both EDV and ESV compared to the spiral real-time CMR and echocardiographic methods, leading to an important overestimation of the ejection fraction (41% vs. 22% for the echocardiographic and spiral CMR method in the first case, and 45% vs. 42% for the second patient). Nevertheless, this underestimation of EDV and ESV using segmented CMR has been observed not only with these two outliers, but also with four other patients for EDV and six patients for ESV, meaning 63% of the included arrhythmic participants.

**Figure 6:**
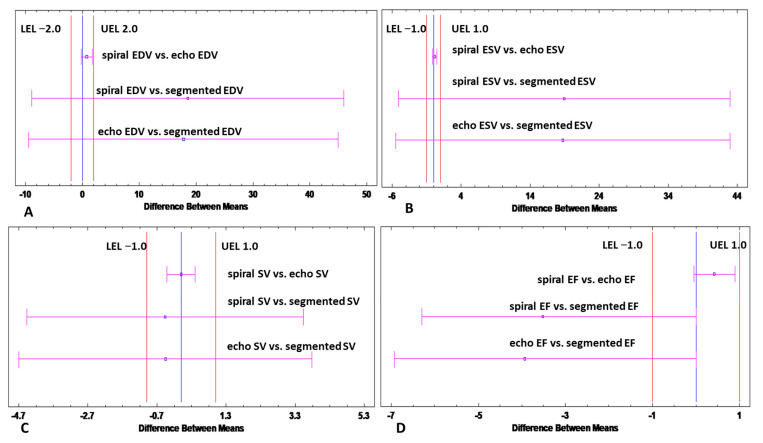
Results of Equivalence analysis. Equivalence analysis demonstrates that means obtained with the spiral method are equivalent to the means assessed by the reference echocardiographic method for (**A**) EDV (**B**) ESV, (**C**) SV, and (**D**) EF. The 95% confidence interval for the difference between the means depicted by the red lines is entirely within the margins of equivalence defined for each parameter. In contrast, for these margins of equivalence, the analysis revealed no equivalence between segmented acquisitions and the reference echocardiographic method. Equivalence test alpha = 5%. Echo = echocardiographic, EDV = end-diastolic volume, EF = ejection fraction, ESV = end-systolic volume, SV = stroke volume, LV = left ventricular, LEL = lower limit of equivalence, ULE = upper limit of equivalence.

Additional two-sample equivalence tests were performed among arrhythmic patients to reinforce the regression results by determining whether the means of either the segmented method or spiral method are equivalent to the means assessed by the reference echocardiographic method. Equivalence was demonstrated for spiral acquisition, while it was not demonstrated for segmented Cartesian acquisition, in EDV (limits of equivalence −2,2), ESV (limits of equivalence −1,1), SV (limits of equivalence −1,1) or LVEF (limits of equivalence −1,1) by showing that the 95% confidence interval for the difference between the means is entirely within the margins of equivalence, for the “spiral vs. echo” case, as shown in Figure 6.

## 4. Discussion

Reliable LV volumetric assessment using standard Cartesian CMR remains a challenge in patients with underlying arrhythmia. In this work, we present a real-time acquisition technique based on spiral k-space sampling combined with compressed sensing. The main findings of this study were that: (i) real-time b-SSFP imaging using spiral trajectories was feasible; (ii) there was good agreement for quantification of biventricular volumetry between the spiral real-time and standard segmented technique in patients with sinus rhythm; (iii) in arrhythmic patients, segmented Cartesian CMR showed significant underestimation of LV-ESV and significant overestimation of LVEF when compared to a reference echocardiographic technique; (iv) real-time spiral volumetry yielded strong agreement with and equivalent relation to volumetry obtained with the reference echocardiographic acquisitions in patients with arrhythmia.

Various applications of spiral k-space sampling in cardiac MRI have been presented in the past [18,19,20]. However, imperfections in the gradient dynamics, resulting in spatial and temporal blurring, have limited clinical applications [21]. Moreover, neither of these sequences has been validated in patients with arrhythmia thus far.

Our study showed that retrospective ECG-gated real-time CMR based on a spiral acquisition technique at 1.5 T had strong agreement with a standard segmented Cartesian-based sequence for biventricular volumetric assessment in patients with sinus rhythm. As reported in other studies, the main limitation of real-time techniques based on non-Cartesian trajectories with b-SSFP readouts relates to poor temporal resolution due to off-resonance effects and longer repetition times [19,22,23,24]. To improve temporal resolution in this case, high acceleration factors were needed and compressed sensing was used for image reconstruction. Spiral k-space filling combined with compressed sensing maximized the temporal resolution and rendered a slightly lower spatial resolution compared to standard segmented acquisition in patients with sinus rhythm. However, in the arrhythmic population, this imaging technique significantly reduced blurring and mis-triggering artifacts, leading to significantly better endocardial delineation. Previous work demonstrated that mainly the temporal resolution and not the spatial resolution determines robust ventricular volumetry in real-time CMR imaging with non-Cartesian sampling [25]. Kaji et al. [12] recommended an effective temporal resolution of ~60 ms for accurate ventricular volume assessment in real-time CMR. The temporal resolution of the spiral real-time sequence in the present study was 39 ms, which was close to the temporal resolution of Cartesian imaging allowing for accurate delineation of the ventricular endocardial borders and strong agreement with the reference standard method. However, a slight but not significant underestimation of LV-EDV was noted using the real-time sequence. These results are in line with previous work [3,14,22] and might rely on the slightly poorer spatial resolution of real-time CMR sequence of 2 × 2 mm^2^ versus 1.4 × 1.4 mm^2^ for the standard cartesian CMR protocol. However, given the good agreement between the volumetry achieved with spiral real-time and standard CMR, the spatial resolution of the real-time CMR sequence does not have clinical implications.

### Clinical Utility in Arrhythmic Patients

The main goal of this study was to assess the performance of the real-time spiral sequence in patients with arrhythmia, where standard segmented CMR often fails to deliver robust volumetry. Metrics obtained with both CMR methods were compared to those assessed by an echocardiographic reference method. This objective quantitative approach is one strength of the current study, since previous and scarce data rely only on subjective qualitative scoring with inherent observer bias [26,27]. On the other hand, we acknowledge the possible measurement discrepancies that might emerge from different modalities; however, most studies have reported good agreement between CMR and echocardiographic LV volumetric assessment [28,29].

The consistency of cardiac motion is pivotal for diagnostic qualitative imaging in standard segmented CMR. Therefore, irregular heartbeats might severely compromise volumetric assessment. The effect of mis-triggering related to arrhythmia is however unlikely to influence real-time CMR imaging, since all data for one slice were generated within one R–R interval.

Indeed, we confirm in the present study superior image quality of the real-time CMR in arrhythmic patients in comparison to standard segmented CMR, allowing for reliable endocardial delineation. Standard segmented CMR showed significant underestimation of LV-ESV and significant overestimation of LVEF when compared to the reference echocardiographic technique in patients with irregular heartbeats. These results reflect the unreliable endocardial contouring with standard Cartesian imaging in arrhythmia, since segmented Cartesian CMR usually generates systematic volumetric overestimation when compared with echocardiography. In contrast, spiral real-time CMR yielded equivalent results with the reference echocardiographic acquisitions, as only minor, statistically non-significant overestimation of EDV could be observed. Previous studies which compared standard segmented CMR volumetry with echocardiographic volumetry demonstrated good agreements between the two methods in patients with sinus rhythm, despite overestimation of metrics obtained with CMR [28,29]. However, the volumetric overestimation observed with real-time CMR compared to echocardiography in this study was minor, and clinically not significant. Lee, et al. [30] demonstrated accurate volumetric assessment with real-time CMR in tachycardic patients (heart rate over 90 bpm) only when the temporal resolution was significantly improved below 90 ms. The excellent temporal resolution in the present study might explain the measurement accuracy and equivalence with the reference method in arrhythmic and tachycardic subjects.

## 5. Limitations

A limitation of this study was the small sample size in a single center; however, it is comparable to previous work and the proportion of arrhythmic patients is superior to other studies that focused on volumetric accuracy in clinical practice [26,31]. Nevertheless, the performance of this real-time sequence should be further optimized in future larger studies. Second, we acknowledge the potential measurement discrepancies that might emerge when comparing an echocardiographic to an MRI approach; the goal of this study was, however, to validate the real-time sequence for LV volumetry in arrhythmic patients using an objective comparison to a reference method, less susceptible to motion artifacts, avoiding the observer bias related to subjective scoring mostly used in previous work. Since there is no validated CMR sequence for accurate volumetric assessment in arrhythmic patients, we considered echocardiography as reference, given that most studies have reported good agreement between the two modalities. We did not perform a similar approach for the RV volumetry assessment in the arrhythmic population, given that a robust comparison of CMR volumetry to a standardized echocardiographic volumetry of the right ventricle is more challenging. Moreover, contrary to the LV, no sufficient data support a good agreement between CMR and echocardiographic RV volumetry. However, the performance of this sequence will be further investigated in future projects focusing on patients with right heart disease. Nevertheless, in patients with sinus rhythm, good agreements were demonstrated between real-time and segmented Cartesian RV volumetry. Finally, more work is required to reduce reconstruction times in real-time CMR to that of standard Cartesian imaging.

## 6. Conclusions

In conclusion, we validated a spiral real-time b-SSFP sequence at 1.5 T, which enabled the assessment of biventricular volumes with a high degree of accuracy in subjects with sinus rhythm. In patients with underlying arrhythmia, compared to the segmented Cartesian imaging, spiral real-time CMR yielded superior image quality and better agreement with the reference echocardiographic volumetry. Therefore, we consider that this real-time sequence could be successfully applied in the arrhythmic population.

## Figures and Tables

**Table 1:** Detailed Parameters of Sequences.

Parameters	Cartesian Segmented CMR	Real-Time Spiral CMR
Sequence type	2D cine b-SSFP	2D cine b-SSFP
TR (ms)	2.42	5.73
TE (ms)	1.2	1.11
Flip angle (°)	60	70
FOV (mm^2^)	380 × 380	340 × 340
Thickness (mm)	8	8
Number of slices	11–17	11–17
In plane resolution (mm^2^)	1.4 × 1.4	2 × 2
Temporal resolution (ms)	32 Phases *	39.9 ms
Time per slice (s)	4 (3 Slices/BH)	3
ECG Mode	Retrospective	Retrospective
Acceleration factor	1.7 (SENSE)	5 (CS with TV regularization)

* Parameter varied depending on heart rate, BH = breath-hold, b-SSFP = balanced steady-state free precession, ECG = electrocardiography, FOV = field of view, TE = echo time, TR = repetition time, 2D = two-dimensional.

**Table 2:** Patients’ demographic and clinical characteristics.

	All (*n* = 32)	Sinus Rhythm (*n* = 21)	Arrythmia (*n* = 11)
Age, years	57 ± 16	55 ± 18	61 ± 12
Male (%)	19 (59.4)	12 (57.1)	7 (63.6)
BMI (kg/m^2^)	26.34 ± 2.06	26.11 ± 1.94	26.78 ± 1.71
BSA (m^2^)	1.99 ± 0.18	1.99 ± 0.18	1.99 ± 0.17
HR (beats/minute) *	72 ± 11	70 ± 11	77 ± 8
Cardiovascular risk factors			
Hypertension	16 (50)	11 (52.4)	5 (45.5)
Hypercholesterolemia	20 (62.5)	13 (61.9)	7 (63.6)
Diabetes	6 (18.8)	5 (23.8)	1 (9.1)
Smoking	14 (43.8)	9 (42.9)	5 (45.5)
CMR-Diagnosis			
Coronary artery disease	11 (34.4)	7 (33.3)	4 (36.4)
Dilated Cardiomyopathy	1 (3.1)	0 (0)	1 (9.1)
Hypertrophic Cardiomyopathy	1 (3.1)	1 (4.8)	0 (0)
Valvular Disease	2 (6.3)	1 (4.8)	1(9.1)
Myocarditis	4 (12.5)	3 (14.3)	1 (9.1)
Other	8 (25)	5 (23.8)	3 (27.2)
Normal finding	5 (15.6)	4 (19)	1 (9.1)
Arrythmia (%)	11 (34.4)	0	11 (100)
Atrial Fibrillation	9 (28.2)	0	9 (81.8)
Ventricular premature beats	2 (6.2)	0	2 (18.2)

* Heart rate during examination.

**Table 3:** Euro-CMR Scores for Image quality for patients in sinus rhythm.

	Segmented Cine	Spiral Real Time	*p*-Value
Wrap-around	0 ± 0	0 ± 0	
Respiratory ghost	0 ± 0	0 ± 0	
Cardiac ghost	0 ± 0	0.1 ± 0.3	0.15
Blurring/mis-triggering	0 ± 0	0 ± 0	
Metallic artifact	0 ± 0	0 ± 0	
Shimming artifact	0.2 ± 0.4	0 ± 0	0.08
Total scoring	0.14 ± 0.35	0.09 ± 0.3	0.64

Euro-CMR = European CMR Registry.

**Table 4:** LV and RV Volume Measurements for patients in sinus rhythm.

	Segmented Cartesian CMR	Real-Time Spiral CMR	*p*-Value
Left ventricle			
LV-EDV (mL)	137.15 ± 31	135.95 ± 30	0.164
LV-ESV (mL)	63.90 ± 20	63.76 ± 19	0.883
LV-SV (mL)	72.42 ± 16	72.19 ± 15	0.818
LV-EF	54.6 ± 6.4	54.00 ± 6.4	0.412
Right ventricle			
RV-EDV (mL)	140.19 ± 31	140.95 ± 31	0.350
RV-ESV (mL)	64.33 ± 20	65.66 ± 19	0.122
RV-SV (mL)	75.80 ± 16	75.19 ± 17	0.522
RV-EF	54.67 ± 6.3	54.04 ± 7.0	0.399

CMR = cardiovascular magnetic resonance, EDV = end-diastolic volume, EF = ejection fraction, ESV = end-systolic volume, LV = left ventricular, RV right ventricular, SV = stroke volume.

**Table 5:** Results of the Bland–Altman Analysis in patients with sinus rhythm.

	LV-EDV	LV-ESV	LV-SV	LV-EF
Mean difference ± SD	1.19 ± 3.77	0.14 ± 4.38	0.23 ± 4.67	0.67 ± 2.71
Limits of agreement	−6.21–8.59	−8.45–8.73	−8.93–9.40	−4.64–5.97
95% Confidence interval	−0.52–2.90	−1.85–2.13	−1.89–2.36	−0.57–1.90
*p*-value	0.164	0.883	0.818	0.273
SE of the mean difference	0.82	0.95	1.02	0.59
Regression line	y = 0.041x − 4.36	y = 0.066x − 4.099	y = 0.061x − 4.176	y = 0.026x − 0.739
*p*-value, mean coefficient	0.141	0.200	0.393	0.800
	RV-EDV	RV-ESV	RV-SV	RV-EF
Mean difference ± SD	−0.76 ± 3.64	−1.33 ± 3.78	0.61 ± 4.35	0.38 ± 3.79
Limits of agreement	−7.90–6.38	−8.75–6.08	−7.91–9.15	−0.07–0.07
95% Confidence interval	−2.42–0.89	−3.05–0.39	−1.36–2.60	−1.34–2.10
*p*-value	0.350	0.122	0.522	0.650
SE of the mean difference	0.79	0.82	0.95	0.83
Regression line	y = −0.01x + 0.576	y = −0.019x − 0.130	y = −0.014x + 1.645	y = 0.036x + 2.052
*p*-value, mean coefficient	0.723	0.680	0.827	0.30

Comparison of real-time CMR and segmented CMR data shows no significant degree of directional measurement bias for any measured LV or RV parameter. No proportional bias is indicated by the linear regression, with no mean coefficient (i.e., slope) significantly different from 0, according to the results of the table. EDV = end-diastolic volume, EF = ejection fraction, ESV = end-systolic volume, SV = stroke volume, LV = left ventricular, RV = right ventricular, SD = standard deviation, SE = standard error.

**Table 6:** Euro-CMR Scores for Image quality in patients with arrhythmia.

	Segmented Cine	Spiral Real Time	*p*-Value
Wrap-around	0 ± 0	0 ± 0	
Respiratory ghost	1.18 ± 1.16	0 ± 0	0.003
Cardiac ghost	1.18 ± 1.16	0.18 ± 0.4	0.014
Blurring/mis-triggering	2.09 ± 0.94	0.09 ± 0.30	<0.001
Metallic artifact	0 ± 0	0 ± 0	
Shimming artifact	0.27 ± 0.64	0 ± 0	0.19
Total scoring	4.72 ± 2.93	0.27 ± 0.64	<0.001

Euro-CMR = European CMR Registry.

**Table 7:** LV Volume Measurements for arrhythmic patients using segmented Cartesian CMR, real-time spiral CMR, and reference echocardiography.

	Segmented Cartesian CMR	Reference Echo	*p*-Value
LVEDV (mL)	177.11 ± 69.98	194.85 ± 75.47	0.264
LVESV (mL)	106.72 ± 63.51	125.47 ± 72.41	0.026
LVSV (mL)	70.40 ± 16.03	69.95 ± 12.90	0.849
LVEF (%)	42.96 ± 10.81	39.02 ± 11.72	0.039
	**Real-time spiral CMR**	**Reference Echo**	***p*-value**
LVEDV (mL)	195.74 ± 76.66	194.85 ± 75.47	0.174
LVESV (mL)	125.64 ± 72.10	125.47 ± 72.41	0.328
LVSV (mL)	69.11 ± 12.70	69.95 ± 12.90	0.976
LVEF (%)	39.44 ± 11.80	39.02 ± 11.72	0.139

CMR = cardiovascular magnetic resonance, Echo = echocardiography, EDV = end-diastolic volume, EF = ejection fraction, ESV = end-systolic volume, LV = left ventricular, SV = stroke volume.

## Data Availability

The datasets used and/or analyzed during the current study are available from the corresponding author on reasonable request.
